# A Case of Spondylodiscitis Fistulating Into the Vagina 15 Years after Promontofixation

**DOI:** 10.5334/jbsr.2699

**Published:** 2022-05-04

**Authors:** Thomas Saliba, Iulia Mocanu, Sanjiva Pather

**Affiliations:** 1 ULB, BE; 2 CHIREC Braine-L’Alleud, BE

**Keywords:** promontofixation, infection, hysterectomy, spondylodiscitis, S. constellatus

## Abstract

Promontofixations can be a rare cause of spondylodiscitis due to the material used getting infected. We present here a case of a 75-year-old woman who underwent a subtotal hysterectomy, followed by a trachelectomy, and presented 15 years later with lumbago and fever. After thorough examination, haemocultures and imaging were performed. This led to the diagnosis of spondylodiscitis of L5-S1, likely due to S. constellatus, with a fistula into the vagina. The patient received surgical treatment. This case is unusual due to the time lapse between the hysterectomy and the infection as well as the probable pathogen.

**Teaching Point:** Promontofixation material can remain despite hysterectomy and can be a source of infection many years after the operation has taken place.

## Introduction

Promontofixation, otherwise known as sacrocolpopexy, is the surgical fixation of the vagina to the sacral promontory to treat genital prolapses and the subsequent urinary incontinence. This is done by suspending the vagina and uterus, returning it to its physiological position. A wide variety of techniques exist, requiring the use of a foreign material (generally mesh or sutures), though, other techniques do exist [1]. Promontofixation patients often complain of lumbago, thought to result from the fixation material, infection, or non-specific inflammation [2,3].

Owing to the operation technique, involving fixation to the sacral promontory, there is the possibility of rare infectious complications of the prosthetic material extending from the genitourinary tract to the L5-S1 disk, causing spondylodiscitis [2]. Once an infection is detected, one can surgically remove the infected foreign body and treat with ad hoc antibiotics [2,4].

## Case History

A 75-year-old woman presented to the emergency room (ER) complaining of a week-long history of lumbago and a three-day history of fever (38.9°C). The pain was described as originating from the thoracic spine, before descending into the lower back. The previous week a non-contrast computed tomography scan (NCCT) was performed to investigate the patient’s pain, establishing a diagnosis of an L5-S1 herniated disk. A hospitalisation for pain management was recommended. She declined and returned home before presenting to our ER the following week.

The patient revealed that she had been experiencing brown vaginal discharges over the last year, which went untreated. She had also undergone a promontofixation 15 years previous, followed by a subtotal hysterectomy two years later, with a trachelectomy the following year.

During the physical examination, the patient reported pain upon palpation of the lumbar spine, irradiating bilaterally into the anterior side of the thighs. She did not complain of hypoesthesia or weakness, but she had difficulty standing and walking due to the pain.

Blood samples were taken and haemocultures performed, revealing an inflammatory syndrome (WBC: 15,000/mm^3^, CRP 226mg/L) as well as a bloodborne multi-sensitive Streptococcus constellatus infection.

The patient was admitted for pain management and suspected infection, pending further investigation of her lumbago. She received treatment by amoxicillin followed by ceftriaxone.

Upon admission, a spinal NCCT was performed (***Figures 1*** and ***2***), followed by a whole-body PET-CT (***Figures 3*** and ***4***), contrast spinal CT (***Figure 9***) and lumbar gadolinium-enhanced MRI (***Figures 5, 6, 7, 8, 10***), leading to the diagnosis of an abscessed spondylodiscitis, fistulising into the vagina.

**Figure 1:**
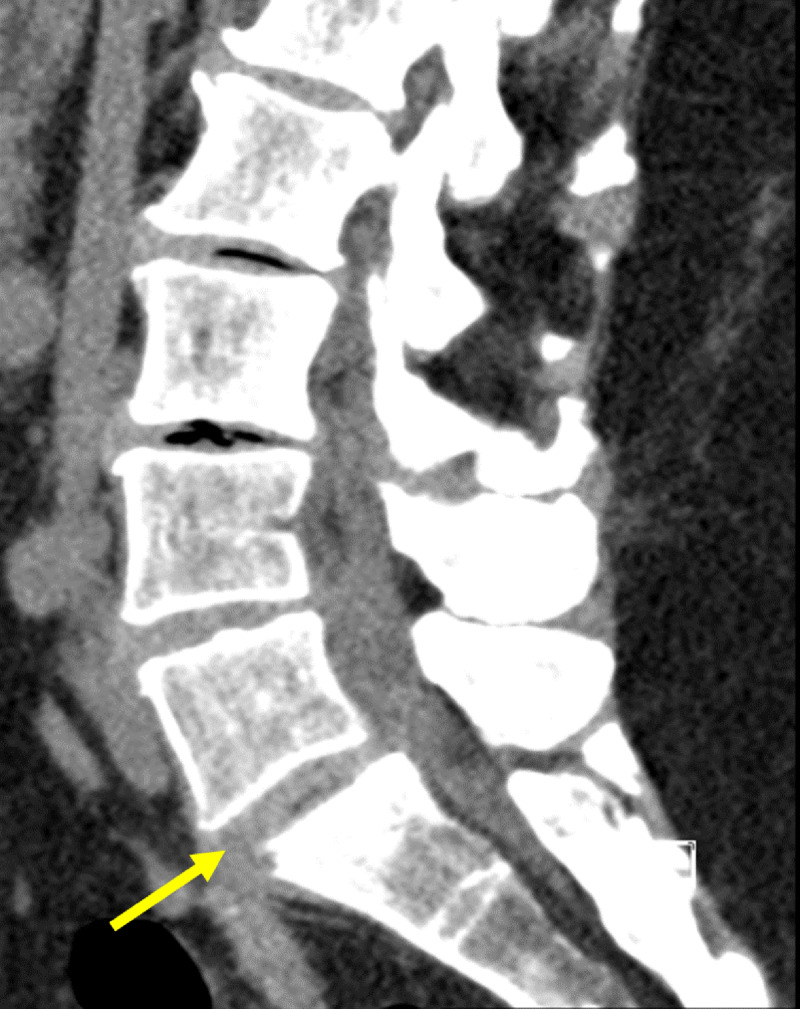
Sagittal non-contrast CT of lumbar spine: Initial CT-scan showing collection from L5-S1 disk with fistulation downwards, an anterolisthesis of L5-S1 with inter-apophysary posterior arthritis and a compression of the right root of S1 by the intervertebral disk.

**Figure 2:**
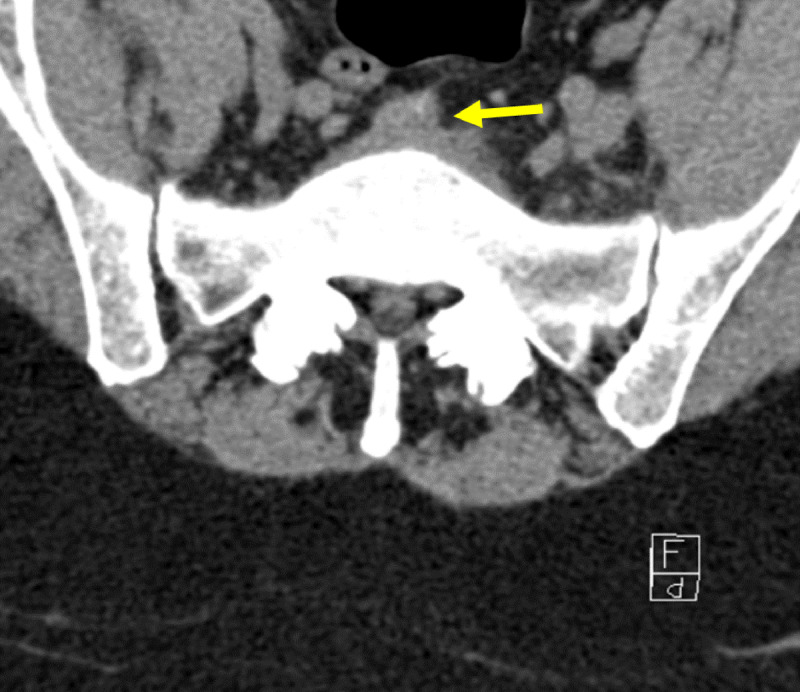
Transverse non-contrast CT of L5-S1 disk: Initial CT-scan showing collection in front of disk.

**Figure 3:**
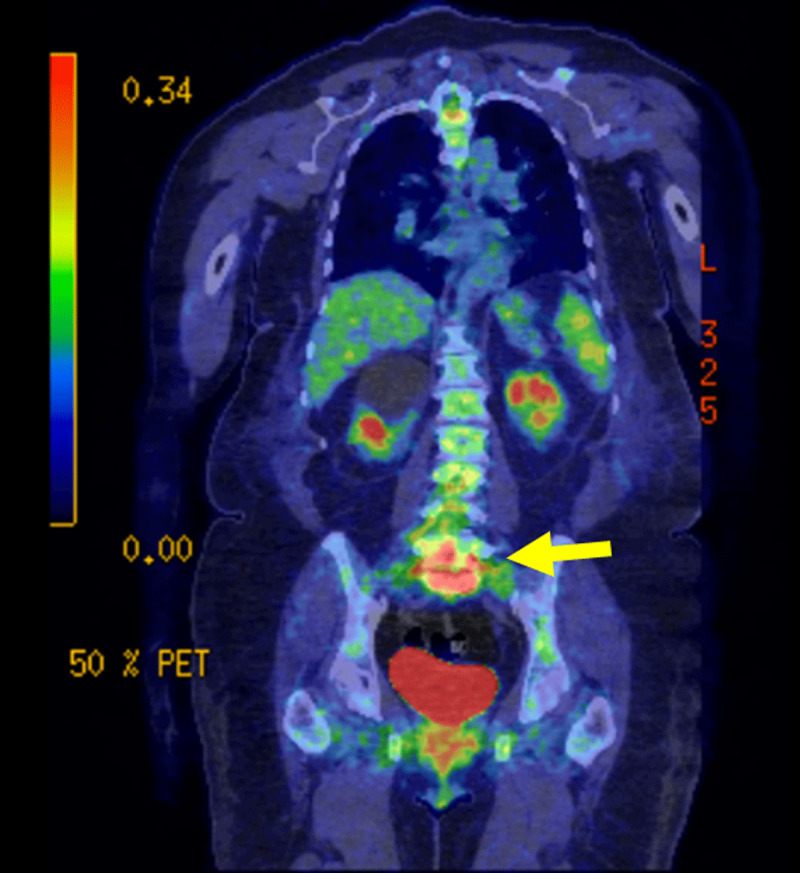
Coronal FDG PET-CT: PET-CT using F-FDG was performed in the context of a possible infection which revealed intense activity at the junction of L5-S1 and the surrounding tissue.

**Figure 4:**
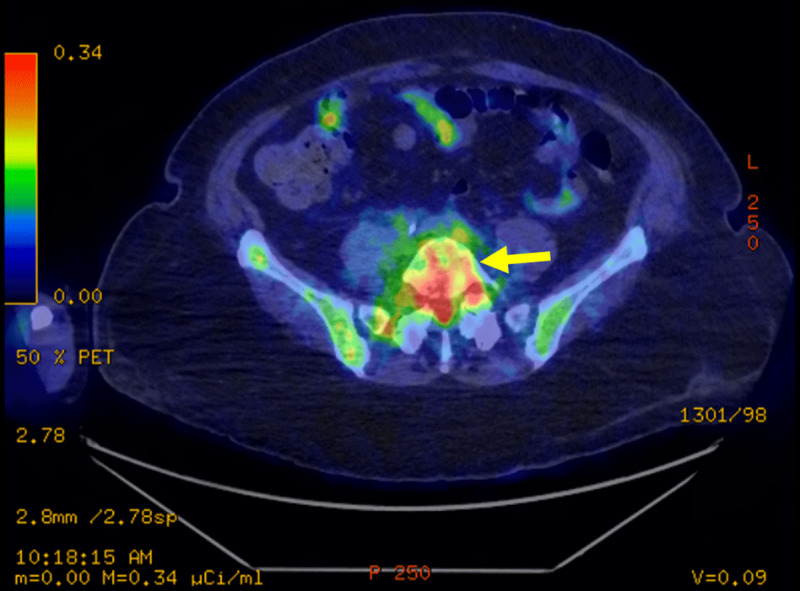
Transverse FDG PET-CT: PET-CT showing hyperfixation of L5-S1 disk.

**Figure 5:**
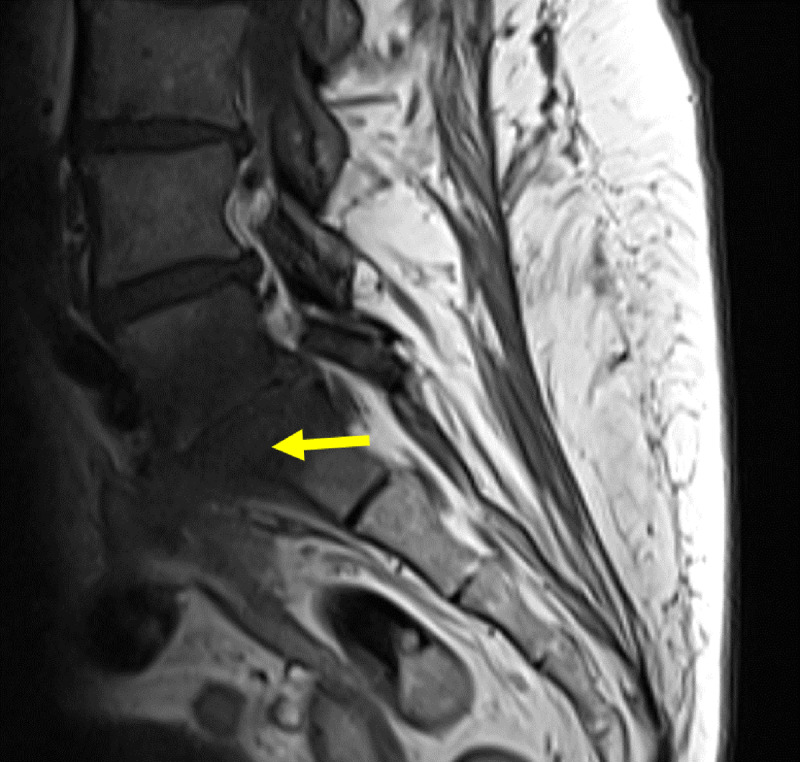
Sagittal contrast MRI in T1 weighting: A medullar bone oedema is seen on either side of the L5-S1 disk. Small collections surround L5-S1 with the largest being 20mm in diameter located on the left psoas muscle with a wall that was intensified by the contrast. This abscess extends posteriorly to the anterior peridural space, the lumbar vertebral bodies, and the last thoracic vertebra.

**Figure 6:**
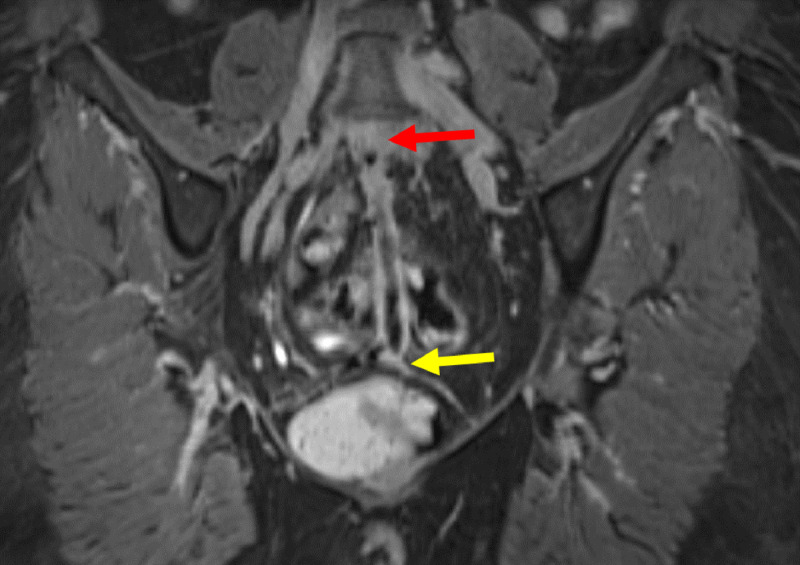
Coronal 3D contrast MRI in T1 fat saturation weighing: Full path of fistula from L5-S1 (yellow arrow) to the vagina (red arrow). The fistula presents with a hyperintense wall surrounding a hypointense lumen.

**Figure 7:**
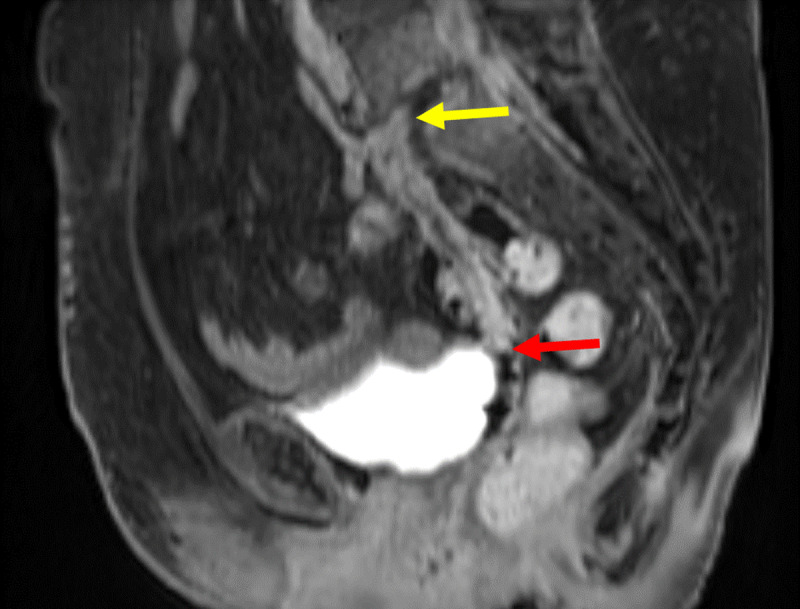
Sagittal contrast MRI in T1 fat saturation weighing: Full path of fistula from L5-S1 (yellow arrow) to the vagina. The fistula (red arrow) presents with a hyperintense wall surrounding a hypointense lumen.

**Figure 8:**
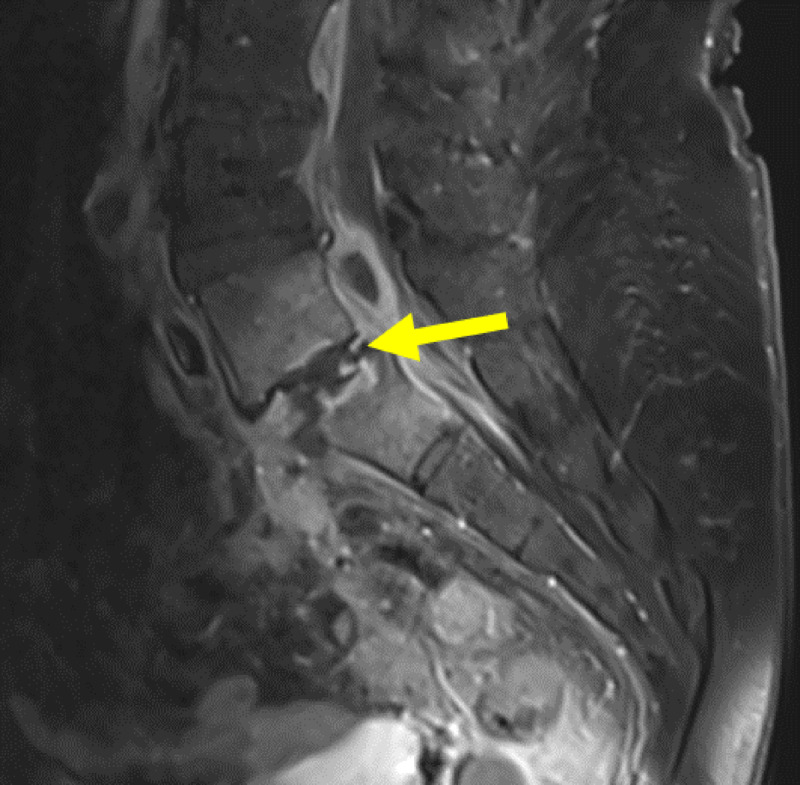
Sagittal contrast MRI in T1 fat saturation weighing: Hyperintense L5-S1 vertebrae and surrounding tissue, showing spondylodiscitis (yellow arrow). There is slight anterolisthesis of L5 upon S1.

**Figure 9:**
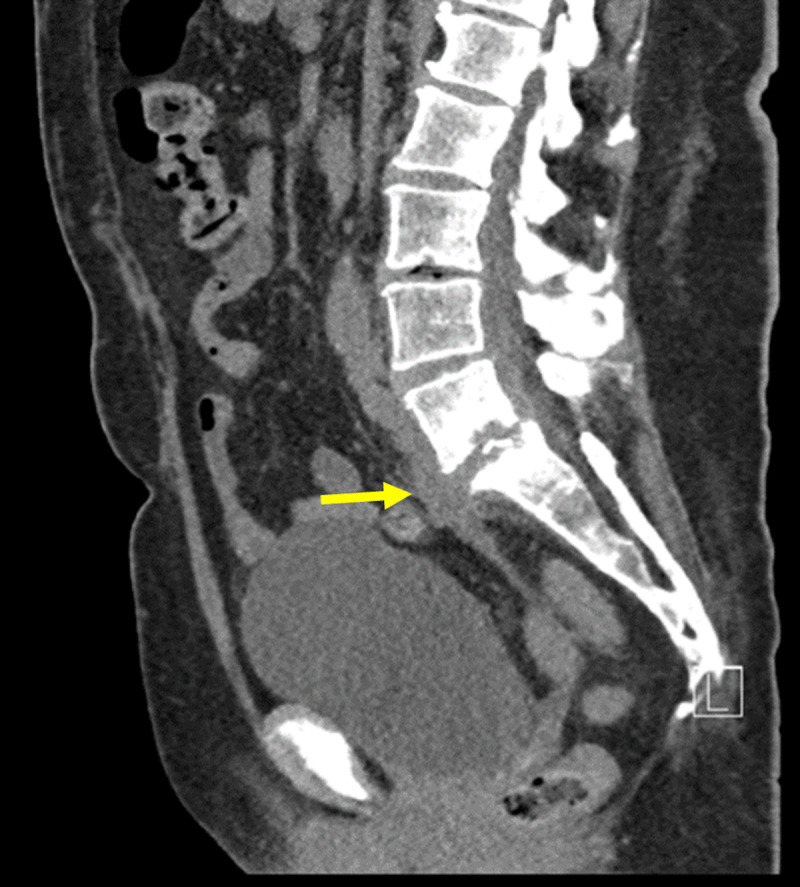
Sagittal contrast CT: Contrast CT-scan showing spondylodiscitis of L5-S1, with infiltration into the soft tissue surrounding S1. Additionally, a 9.7cm fistula can be seen, starting at the intervertebral disk of L5-S1 (yellow arrow) to the vagina.

**Figure 10:**
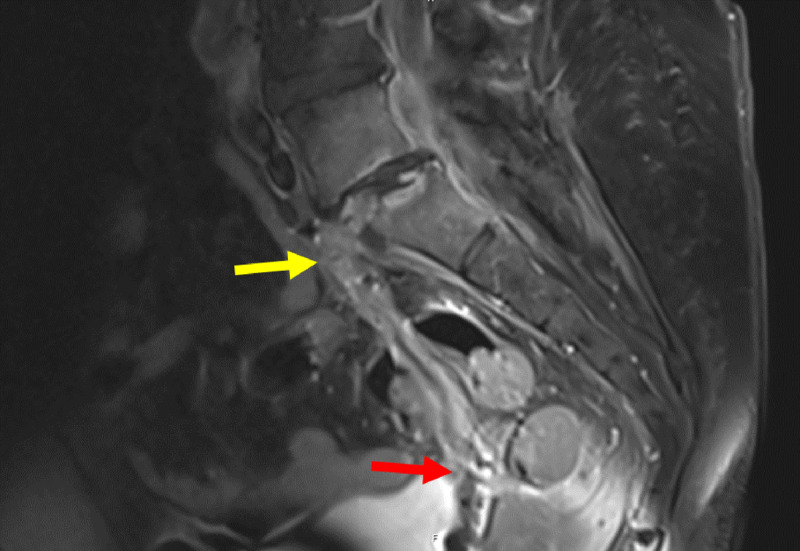
Sagittal contrast MRI in T1 weighting: Pre-operative MRI showing an epidural collection behind the vertebral body of L5 and within the L5-S1 disk (yellow arrow), fistulising into the vagina (red arrow).

The pre-operative MRI revealed the path of the fistula (***Figure 8***). Laparoscopic surgery was performed to treat the ascending spondylodiscitis and investigate the cause of the infection. The pre-vertebral abscess was drained, and the fistula was repaired. Samples were sent to pathology. Analysis of the samples revealed fragments of the material used in the promontofixation, though bacteriological analysis was negative.

## Comment

We presented here a case of spondylodiscitis caused by infected promontofixation material in a patient who had undergone a sub-total hysterectomy and trachelectomy 15 years previously.

During her workup, a gynaecological examination was conducted. The hypothesis was that the infection resulted from remaining promontofixation material, which was not removed when the subtotal hysterectomy, and trachelectomy, was performed. However, colonisation of the material post-septicaemia was also considered.

Infections related to promontofixation are relatively rare, with varying infection rates according to the surgical method used [3,4]. Infection occurs in 3.9% of laparoscopic promontofixations, 3.8% of robot-assisted laparoscopic promontofixations, and 10.8% of abdominal promontofixations [3]. In cases of post-promontofixation spondylodiscitis, the pathogen was found in 77% of cases [5].

Spondylodiscitis can be identified using many imaging modalities [6]. A non-contrast CT-scan will show a loss of disk height, end plate irregularities, erosion, and swelling of the paravertebral soft tissues [6]. Magnetic resonance imaging (MRI) has a sensitivity of 97%, specificity of 93%, and an accuracy of 94% in diagnosing spondylodiscitis, making it the gold standard imagery [5,6]. An MRI with both T1 and T2 contrast images is required, as well as T1 sequences with fat suppression [6]. The inflammation will present as zones of hypo or isointense T1 and a hyperintense T2 [6]. The signal changes are often seen on the anterior aspect of the vertebral body with a single segment involved [6]. Gadolinium contrast will enhance the subchondral bone, with diffuse enhancement of the affected disk, allowing the identification of an abscess within the disk or bone [6].

Positron emission tomography CT (PET-CT) using ^18^F-FDG is highly specific for infectious spondylodiscitis, with some studies reporting up to 100% accuracy in their diagnoses [6].

The particularity of this case is that the spondylodiscitis occurred 15 years after the promontofixation and hysterectomy. As most complications appear within the first year, our case is a rare occurrence [2,5].

Another interesting feature of this case is the presumed infectious agent, Streptococcus constellatus, isolated in the haemoculture. This rarely causes spondylodiscitis, with only two other cases reported in the literature [7]. This is compounded by the fact that S. constellatus is rarely found in abscesses involving the genito-urinary tract which, due to the path of the fistula, is the presumed route of infection in this case [8]. The examination of the intra-operative samples taken from the spondylodiscitis by the pathologist and the pus by the lab, no infectious agent was discovered. This may be due to the use of antibiotics prior to the operation. This is in keeping with the literature, wherein a causative agent is not always located on the sample after prior antibiotic treatment for the spondylodiscitis [2,4].

It is notable that our patient underwent a hysterectomy after her promontofixation, as it has been shown that hysterectomy increases the risk of mesh exposure, as may have been the case here [1]. Furthermore, though subtotal hysterectomy, which our patient underwent at first, results in a reduced rate of mesh exposure, the subsequent trachelectomy increased the risk of infection for the patient [1].

## Conclusion

Promontopexy is generally well tolerated and infrequently causes infection. Our case shows a rare cause of spondylodiscitis, fistulising along the path of the promontopexy material, with a positive S. constellatus haemoculture. This case is also unusual because of the time lapse between the operation infectious episode due to the patient having undergone iterative gynaecological operations after the promontopexy.
